# On the Value of Widal's Reaction

**Published:** 1899-12-09

**Authors:** J. O. Symes

**Affiliations:** Bacteriologist, Bristol Royal Infirmary.


### Hospital Clinics and Medical Progress.

### ON THE VALUE OF WIDAL'S REACTION.

By J. O. Symes, M.D., D.P.H., Bacteriologist, Bristol Royal Infirmary.

The discovery by Widal and Grünbaum in 1896 that the sera of patients suffering from certain diseases was capable of agglomerating the specific organisms of these diseases, promised at the time to be one of great practical utility.

During the past three years the reaction has been given an extended trial, more especially in connection with the diagnosis of enteric fever, and we are now in a position to judge of its value as an aid to diagnosis. The technique of the test is so familiar as to render it unnecessary to describe it in detail in the present paper. It consists essentially of adding the suspected blood, previously diluted, to a young actively motile culture of the typhoid bacillus. If the blood be that of a typhoid patient the bacilli become motionless and agglomerated, whilst in the case of healthy or of diseased persons not affected with typhoid fever no such reaction is obtained.

During the early months of its employment several observers recorded misleading results which had been obtained from this test, agglomeration having been obtained with blood other than that of persons suffering from enteric fever. These errors arose chiefly from two causes—either the blood was insufficiently diluted, and so gave a false partial reaction; or the broth in which the culture was made was too alkaline, and in such a medium the bacilli have but very little movement, and tend to clump spontaneously. The dilution, which was formerly 1 in 10, has now been raised to 1 in 50, or even higher, and at the same time the use of a faintly acid broth medium has been adopted. These and other small changes in the technique of the test have done much to increase its reliability, and at the present time the margin of error is not greater than from 2 to 5 per cent., varying, of course, with various observers.

Our own results have been as follows:—

During the last six months of 1897 40 cases were treated, using a dilution of 1 in 10. Thirty-three positive reactions were obtained, and seven negative. All the cases giving positive reactions were proved either clinically or by post-mortem examination to be enteric fever. Of the seven negative four proved to be cases of enteric fever, and three other diseases. There was thus 10 per cent. of error.

During 1898 71 cases were examined: 20 gave positive results, 49 negative, and two doubtful. Of the 20 positive, all proved clinically, or on post-mortem examination, to be enteric. Of the 49 cases giving negative reactions, 45 were not enteric and four proved to be enteric, in each of which a positive result was obtained some days later. Three of these cases were examined on one day, when the culture was so obviously defective that fresh specimens of blood were sent to the Health Department and positive results were obtained. In the fourth case the blood examined on the eighteenth day of the illness gave no reaction, but on the twentieth day the reaction was positive. Amongst the cases giving negative results were rheumatic fever, pneumonia, diarrhœa, influenza, measles, malaria, meningitis, appendicitis, septicæmia, &c. Of the two doubtful cases one was malaria. Inhibition of movement occurred in these cases, but no "clumping." The dilutions employed varied from 1 in 20 to 1 in 50, and the percentage of error was 5·6.

During the first eight months of 1899, working with dilutions of 1 in 30 and 1 in 50, 40 cases have been tested. Fifteen giving positive reaction were all enteric fever; two, in which the early reactions were doubtful, subsequently proved to be enteric, whilst the 23 negative results were obtained from cases of disease other than typhoid fever. It is thus obvious that we have in Widal's reaction a test of great diagnostic value. At the same time we are not justified in minimising in any way the importance of clinical methods, for from time to time cases of enteric fever occur in which the blood, when tested, fails to give a definite reaction. The two cases returned as doubtful in 1899 are good instances of this fact. In one case the blood was examined on the sixteenth, seventeenth, and twentieth days, and gave no definite reaction; but on the forty-eighth day, during a period of relapse, a good positive result was obtained. In the second case, which terminated fatally, the blood, which was examined three times, never gave a certain result, even during the course of a relapse.

It is extremely difficult to give with certainty the exact date of the commencement of an attack of enteric fever, and consequently one cannot say definitely how early the blood gives this reaction. It is generally to be obtained at the end of the first week of illness. The agglutinating power remains in the blood long after convalescence. We have frequently found it present two to three months after the illness, and in one case obtained a weak reaction after the lapse of two years. A case tested five years after an attack gave no result. The nature and intensity of the reaction is no criterion of the severity of the case, for one frequently gets an instantaneous and complete reaction with the blood of an apparently mild case. At the same time it is worthy of note that both patients from whose blood a doubtful reaction was obtained in 1899 suffered subsequently from relapses. Even when dried the blood retains its agglutinating power, and we have obtained good results from blood stains on paper, on linen, and on other fabrics. The milk, tears, and urine of patients suffering from typhoid fever may also be used for this purpose, but are less reliable than the blood. We have had good reactions from pleuritic fluid taken in the course of enteric fever, and from the blood taken after death. In a case which died pregnant, the blood of the mother gave Widal's reactions, but not that of the fœtus. Heating destroys the agglutinating power, so that it is important to see that the blood is not exposed to too high a temperature when sealing the ends of the pipettes. Widal's reaction is not only of practical service in assisting the diagnosis in a case of supposed enteric fever, it may be sometimes used with advantage in determining what persons in a community have recently suffered from enteric fever. Thus, during a recent epidemic we were able to say of a number of persons who were convalescent from illness of indefinite nature, that they had undoubtedly suffered from mild attacks of typhoid. The test is also employed after anti-typhoid vaccination to ascertain when immunity is secured. Still more useful is its employment in differentiating the B. typhosus from the varieties of B. coli. For instance, in contaminated water, or in suspected stools, there is found a bacillus which may be either of the above; by adding the blood of a typhoid patient to a drop of the living culture we are enabled at once to decide which of the two varieties we are dealing with, for the B. typhosus will be clumped, whilst the B. coli will not. But Widal's reaction is not confined to enteric fever; a like agglomeration occurs with other bacilli when treated with the sera of persons suffering from their associated diseases. Thus cultures of B. melitensis are clumped by the addition of blood from cases of Malta fever, and cultures of Gartner's bacillus by the blood of persons suffering from certain forms of food poisoning. The tubercle bacillus, too, reacts to the blood of persons suffering from tuberculous diseases, but we have found this reaction very imperfect, and of little practical value.

Widal's discovery has brought us a step nearer the possibility of securing an antitoxin for the treatment of enteric fever. Wright's anti-typhoid vaccination is being widely adopted as a prophyllactic measure by persons resident in tropical countries, but the measure of its success has yet to be seen. No curative serum has as yet proved of value.

